# Efficacy of diode Laser compared to conventional irrigation in endodontic treatment of apical periodontitis: a systematic review and meta-analysis

**DOI:** 10.3389/froh.2026.1814056

**Published:** 2026-05-29

**Authors:** Mario G. Casaretto-Gamonal, Victor Moreno-Prieto, Angela M. Murillo-Almache, Nancy Calzada-Gonzales, Oriana Rivera-Lozada, Joshuan J. Barboza

**Affiliations:** 1 Cayetano Heredia University, Lima, Peru; 2 Universidad Nacional Federico Villarreal, Lima, Peru; 3 San Gregorio de Portoviejo University, Portoviejo, Ecuador; 4 Escuela Profesional de Odontología, Universidad Nacional Hermilio Valdizán, Huanuco, Peru; 5 Vicerrectorado de Investigación, Universidad Señor de Sipan, Chiclayo, Peru

**Keywords:** apical periodontitis, conventional irrigation, diode laser, efficacy, endodontic infection, endodontic irrigation, endodontic treatments, healing

## Abstract

**Background:**

Effective disinfection is essential for the successful management of apical periodontitis. Diode lasers have emerged as a promising adjunct to conventional irrigation techniques in endodontics, offering potential benefits in microbial control and postoperative recovery. However, their comparative clinical efficacy remains uncertain.

**Objective:**

This systematic review aims to evaluate the effectiveness and safety of diode laser-assisted irrigation vs. conventional irrigation methods in endodontic treatment of apical periodontitis, focusing on clinical outcomes, radiographic healing, and adverse events.

**Methods:**

A systematic search was conducted in PubMed, Scopus, Web of Science, and Embase following PRISMA 2020 guidelines. Randomized controlled trials comparing diode laser irrigation with conventional methods were included. Three independent reviewers performed study selection, data extraction, and risk of bias assessment using the RoB 2.0 tool. Primary outcomes included bacterial reduction, postoperative pain, and radiographic healing. Secondary outcomes addressed adverse events and procedural complications.

**Results:**

Twelve randomized controlled trials were included, encompassing a total of 863 participants. Diode laser-assisted irrigation demonstrated superior bacterial reduction and postoperative pain control in several studies, particularly when used in conjunction with adjunctive methods such as photodynamic therapy. Radiographic healing outcomes varied across studies, and no serious adverse effects were reported. However, methodological heterogeneity and variability in laser protocols limited comparability.

**Conclusion:**

Diode lasers may provide clinical advantages over conventional irrigation in the management of apical periodontitis, particularly in reducing bacterial load and early postoperative pain. Nevertheless, further high-quality trials with standardized protocols are required to confirm these findings and support their widespread clinical adoption.

**Systematic Review Registration:**

PROSPERO: CRD420251025177.

## Introduction

Apical periodontitis is a chronic inflammatory condition of periapical tissues, typically resulting from persistent microbial infection within the root canal system. Periapical tissue destruction and bone resorption characterize this condition and may lead to progressive tissue damage if not adequately treated (1, 2).

The quality of root canal fillings significantly influences the prevalence of AP (3, 4). Periapical tissue destruction and bone resorption characterize a persistent inflammatory condition, often exacerbated by microbial biofilms and host immune responses (5–7). Conventional endodontic treatments aim to eliminate intracanal pathogens but are frequently hindered by complex root canal anatomy and the presence of resistant species, such as Enterococcus faecalis (6, 8).

Advances in endodontic technology, particularly diode laser-assisted therapies, have shown promise in enhancing disinfection and promoting periapical healing (9–13). Diode lasers operate through thermal and optical mechanisms to penetrate dentinal tubules, disrupt biofilms, and modulate inflammation (14, 15). Laser-assisted endodontic treatments have demonstrated potential benefits, including reduced postoperative pain (10), enhanced antimicrobial efficacy, and accelerated periapical tissue regeneration, compared to conventional methods (16, 17). However, variability in laser parameters and study designs has limited the generalizability of these findings (18).

Recent systematic reviews highlight the need for comparative studies on the clinical and microbiological outcomes of diode lasers in dental treatments (19). Preliminary evidence suggests that diode lasers, especially when combined with adjunctive agents such as sodium hypochlorite (NaOCl) or photodynamic therapy (PDT), are effective in reducing bacterial loads and enhancing radiographic healing (20–24). Nevertheless, concerns regarding safety, such as the apical extrusion of irrigants and thermal damage, necessitate further exploration (25).

Although previous systematic reviews have evaluated the role of laser-assisted disinfection in endodontics, most have focused on isolated outcomes such as postoperative pain or have included heterogeneous clinical conditions without specifically addressing apical periodontitis under controlled comparative settings. Furthermore, recent randomized controlled trials evaluating diode laser protocols have not been fully integrated into earlier evidence syntheses.

Therefore, this systematic review aims to provide an updated and comprehensive evaluation of diode laser therapy as an adjunct to conventional irrigation in patients with apical periodontitis, incorporating newly available randomized evidence and examining multiple clinically relevant outcomes, including antimicrobial efficacy, postoperative pain, radiographic healing, and safety. In addition, this review explores the variability of laser parameters across studies to better understand their potential influence on clinical outcomes.

## Methods

### Study design

This systematic review was conducted and reported in accordance with the Preferred Reporting Items for Systematic Reviews and Meta-Analyses (PRISMA 2020) guidelines. The PRISMA 2020 checklist is provided as Supplementary Table S1.

### Searches

Electronic searches were performed in four databases: PubMed, Scopus, Web of Science, and Embase. The search strategy included terms and controlled vocabularies such as MeSH (for PubMed) and Emtree (for Embase and Scopus), covering studies from the inception of each database up to August 8, 2024. The main search terms were: (“apical periodontitis”) AND (“endodontic treatments”) AND (“diode laser”) AND (“conventional irrigation”). No restrictions were placed on language or publication date. Additionally, the reference lists of relevant studies and previously published reviews were manually screened to identify any additional eligible studies. We added the strategies for each database in Supplementary Table S2.

### Eligibility criteria

This systematic review included clinical trials that met the following eligibility conditions. Only randomized controlled trials (RCTs) with a parallel-group design were considered, provided they involved human participants diagnosed with apical periodontitis undergoing non-surgical endodontic treatment. The population of interest consisted of adult patients with either symptomatic or asymptomatic apical periodontitis.

The intervention evaluated was diode laser-assisted irrigation, applied either as a primary method of disinfection or in conjunction with standard irrigants such as sodium hypochlorite or ethylenediaminetetraacetic acid (EDTA). The comparison group comprised conventional irrigation techniques, including manual irrigation or mechanical activation methods (e.g., sonic or ultrasonic devices), without the use of any form of laser assistance.

To be included, studies had to report at least one relevant clinical outcome related to the efficacy or safety of the irrigation protocol. These outcomes included bacterial load reduction, postoperative pain levels, radiographic healing of periapical tissues, and the occurrence of any adverse events or complications associated with the irrigation technique.

Eligible studies were required to provide sufficient methodological detail regarding their randomization procedures, the nature of the interventions and comparators, and the measurement and reporting of outcomes. Trials that used diode lasers exclusively for purposes other than irrigation activation—such as photobiomodulation without intracanal application—were excluded. Additionally, *in vitro* studies, animal experiments, narrative reviews, systematic reviews, case series, case reports, conference abstracts, editorials, and letters to the editor were not considered for inclusion.

### Exclusion criteria

Studies were excluded if they were conference abstracts, systematic reviews, narrative reviews, case reports, case series, or letters to the editor.

### Outcomes

The primary outcomes assessed included bacterial reduction, postoperative pain reduction, and radiographic healing. Secondary outcomes involved the evaluation of adverse events and procedural complications associated with each irrigation method.

### Data extraction

Following the electronic searches, all identified references were imported into a single library and duplicates were removed. Titles and abstracts were then screened independently by reviewers using predefined inclusion and exclusion criteria through the Rayyan platform. Full-text articles were retrieved and reviewed for eligibility. A second phase of screening was conducted to ensure that all inclusion and exclusion criteria were appropriately applied. Any discrepancies between reviewers were resolved through discussion with a third author (JJB).

Data were extracted independently and in duplicate using a pre-designed Excel spreadsheet. Extracted information included the first author, year of publication, country of origin, study design, sample size per intervention group, inclusion criteria, description of intervention and control groups, and both primary and secondary outcomes.

### Risk of bias assessment

The risk of bias for each included RCT was independently assessed by two reviewers using the Cochrane Risk of Bias 2.0 (RoB 2.0) tool. Any disagreements were resolved through consultation with a third reviewer (JJB). The risk of bias was categorized as low, some concerns, or high, depending on the evaluation of individual domains for each study.

### Data synthesis

Given the expected clinical and methodological heterogeneity among included studies, quantitative synthesis was performed only when sufficient comparability across interventions, populations, and outcomes was identified. The meta-analysis was therefore considered exploratory, and pooled estimates were interpreted with caution. When substantial heterogeneity or high risk of bias was present, findings were primarily summarized using a narrative synthesis approach.

## Results

### Selection of studies

**Figure 1:**
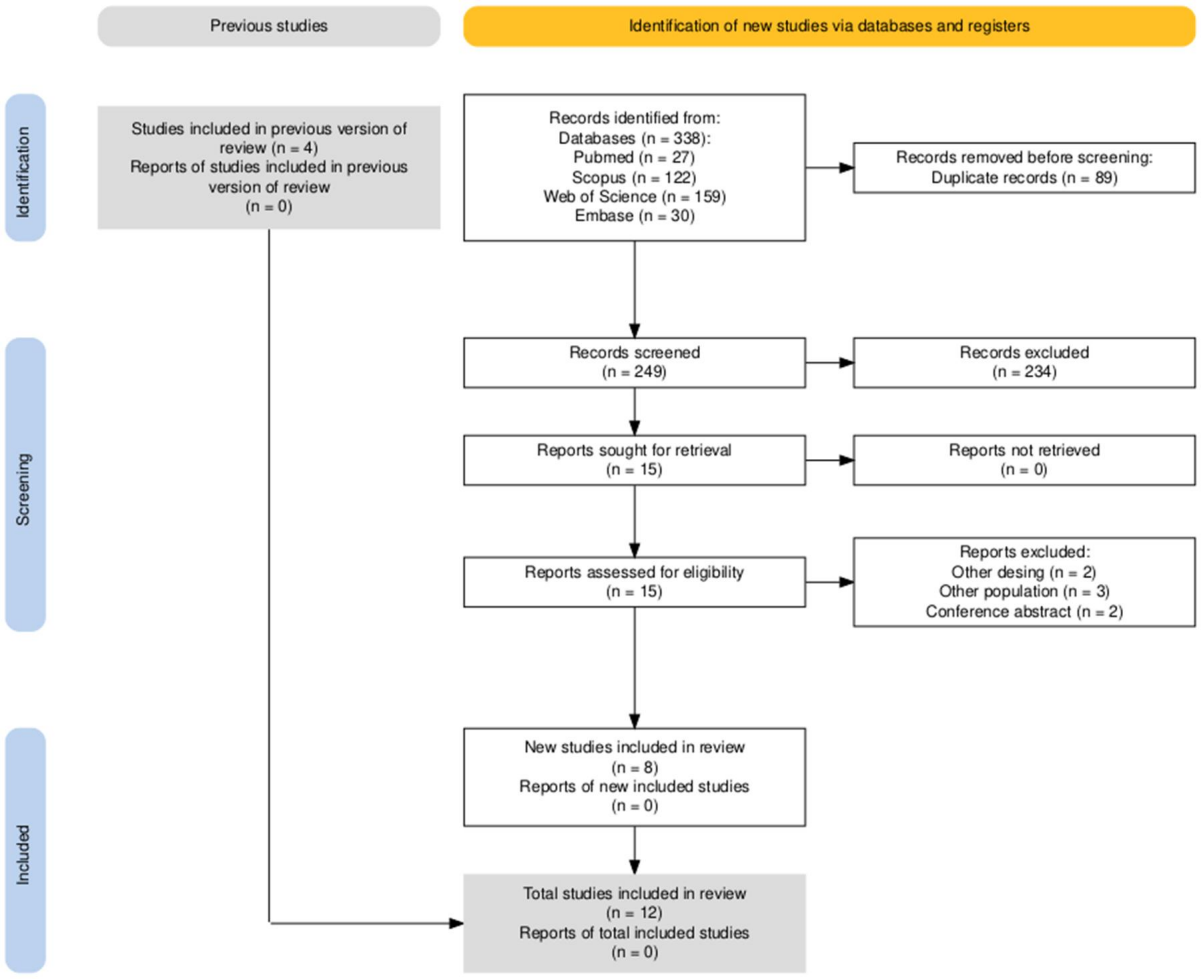
PRISMA 2020 flow diagram of study selection.

Figure 1 shows the PRISMA flow diagram of the identification, screening, exclusion, and inclusion of studies in this systematic review. A total of 338 records were obtained from the databases; 89 duplicate records were removed, 239 reports were excluded using automatic filters or manually after selecting titles and abstracts, and reports were assessed for eligibility. Finally, 12 randomized controlled trials were included (10, 15, 16, 18, 26–29, 31–34).

Due to variability in laser protocols and outcome reporting, not all outcomes were suitable for quantitative synthesis. Therefore, a combination of meta-analysis and structured narrative synthesis was used to present the findings.

### Characteristics of included studies

The characteristics of the studies included in this systematic review reveal a broad diversity in terms of geographical origin, methodologies, and participant profiles. A total of twelve randomized clinical trials (RCTs) conducted in Egypt, Syria, Brazil, India, Turkey, Iran, and Germany were analyzed, representing a wide range of clinical settings. All studies employed a randomized design, with two of them using a triple-blind methodology. The majority of studies utilized a double-blind approach to enhance the reliability of outcomes by minimizing observer and participant biases. In contrast, three studies adopted a single-blind approach, in which only the patient was unaware of the treatment allocation. Most studies provided detailed descriptions of the randomization methods, achieving random allocation through sealed envelopes, predefined study plans, folded paper numbers, or software-generated sequences, ensuring allocation concealment. However, one study did not specify the method of randomization. The total sample size across all included studies is 863 participants. Sample sizes varied from 19 to 180 participants, with patient allocation balanced across intervention and control groups. Follow-up durations demonstrated significant heterogeneity, ranging from short-term evaluations of 72 h to 12 months, with two studies not specifying follow-up periods. Patient ages varied widely across studies, with broader ranges spanning 18 to 65 years. The distribution of sex among patients varied across studies, with some providing explicit details while others lacked demographic specificity. One study reported a total of 32 men and 28 women, while another study described the distribution as 40% men and 60% women.

Additionally, one study included only male participants. Some studies mentioned the inclusion of both men and women, but did not specify exact numbers. Other studies provided more detailed breakdowns: One study reported a 45% male and 55% female distribution, another study specified group distributions: the diode laser group had 14 men and 14 women, while the control group had 10 men and 18 women, a different study detailed the composition of intervention groups: Ca(OH)₂ + Laser Irrigation included 25% men and 75% women, while the Ca(OH)₂ group consisted of 36% men and 66% women, a study on Low-level laser therapy intervention reported eight men and 10 women, while the placebo group consisted of 13 men and five women. The inclusion criteria were consistent in targeting teeth with pulp necrosis and apical periodontitis. Most studies focused on single-rooted teeth, while some included molars with symptomatic or asymptomatic apical periodontitis.

Exclusion criteria frequently addressed potential confounders such as systemic diseases, recent use of antibiotics or analgesics, pregnancy, and specific dental conditions, including open apices or severe periodontal disease. This collection of studies provides a robust foundation for the systematic evaluation of diode laser efficacy compared to conventional irrigation in endodontic treatment. Despite differences in follow-up periods, sample demographics, and study designs, the consistent application of randomized clinical trial methodologies underscores the reliability of the data. This heterogeneity in design and implementation reflects real-world clinical diversity and enhances the generalizability of the findings to varied clinical practices (Supplementary Table S1).

The intervention groups frequently utilized advanced complementary activation techniques, including diode lasers with different wavelength ranges, applied with various power and frequency configurations. Among these, the use of diode lasers ranging from 660 to 980 nm in Laser-Activated Irrigation (LAI) and from 810 to 980 nm in photodynamic therapy (PDT) has been reported. The power employed in diode lasers varied between 0.5 W and 2.5 W, with frequencies in pulsed and continuous modes, depending on the protocol of each study. In the case of LAI, the laser optical fiber was inserted into the root canal to a depth of 1 to 3 mm before the apex, ensuring effective activation of the irrigant without extrusion. Additionally, Er, Cr: YSGG lasers (2,780 nm) were used with a configuration of 1.25 W and 20 Hz, in some cases combined with diode lasers to optimize irrigant activation. Some studies have explored the impact of a 980 nm diode laser in pulsed mode at 1.5 W.

In comparison, others have applied an 810 nm diode laser with a power of 0.8 W and a frequency of 10 Hz, both aimed at improving root canal system decontamination. For photodynamic therapy (PDT), the 810–980 nm diode laser was used in combination with a photosensitizer, typically methylene blue or toluidine blue. Depending on the study protocol, the laser optical fiber was placed in the coronal portion of the root canal, inserted between 2 and 5 mm inside the canal, or, in some cases, applied externally to the buccal and palatal areas at the level of the treated tooth. This technique aimed to facilitate light penetration through tissues, reaching the periapical region and maximizing the activation of the photosensitizer to generate reactive oxygen species with greater antimicrobial effect. In the control groups, ultrasonic and sonic activation techniques were employed, including the EDDY system (6,000 Hz) and Passive Ultrasonic Irrigation (PUI) at 30 kHz for 45 s, which allowed for improved irrigant penetration into the canals. Mechanical activation devices, such as the XP-Endo Finisher, were also incorporated, and the effectiveness of innovative irrigants, including nanochitosan, was evaluated to enhance biofilm removal and reduce bacterial load (Supplementary Table S1).

The results presented in Supplementary Table S1 highlight the variability in the assessment of clinical and radiographic parameters across the included studies. Periapical healing was evaluated in studies using the Periapical Index Score (PAI score), showing a statistically significant reduction in lesion size in groups treated with low-level laser therapy (LLLT) compared to control groups. However, other studies found no significant differences in radiographic healing rates between calcium hydroxide and diode laser treatments, suggesting that both approaches may offer comparable outcomes in periapical regeneration. Postoperative pain reduction was assessed in multiple studies, with some showing statistically significant differences depending on the treatment protocol. Photodynamic therapy (PDT) demonstrated a significantly greater reduction in pain compared to other treatments, suggesting a superior analgesic effect when combined with an appropriate photosensitizer.

Additionally, laser-activated irrigation (LAI) resulted in a statistically significant reduction in pain within the first 48 to 72 h post-treatment, compared to control groups. Similarly, low-level laser therapy (LLLT) was associated with a significant reduction in pain within the first 24 to 72 h, compared to conventional techniques. However, in some studies, the difference between diode laser-treated groups and controls did not reach statistical significance, indicating variability in response depending on laser settings and treatment protocols. Regarding bacterial load reduction, studies suggested that the application of diode lasers, particularly at a 445 nm wavelength, contributed to a statistically significant reduction in bacterial load within the root canals. However, the heterogeneity in assessment methods and measurement time points limits the generalization of these findings. Concerning tooth survival, most studies found no statistically significant differences between laser-treated and control groups, suggesting that diode laser application in endodontics does not negatively impact long-term clinical success rates. In terms of safety, no significant differences were reported in postoperative complications, reinforcing the safety profile of diode lasers in endodontic treatment. No studies reported cases of hypersensitivity, necrosis, or other clinically relevant complications. These findings underscore the importance of accurate laser settings and the necessity of standardized protocols to optimize effectiveness. The presence of statistically significant differences in pain reduction with PDT, LAI, and LLLT, as well as in periapical healing with LLLT, suggests that lasers may be a valuable alternative in endodontic treatment. However, the lack of uniformity in evaluating key outcomes such as radiographic healing and pain reduction underscores the need for additional studies with more standardized methodologies to draw stronger conclusions (Supplementary Table S1).

### Risk of bias assessment

**Figure 2:**
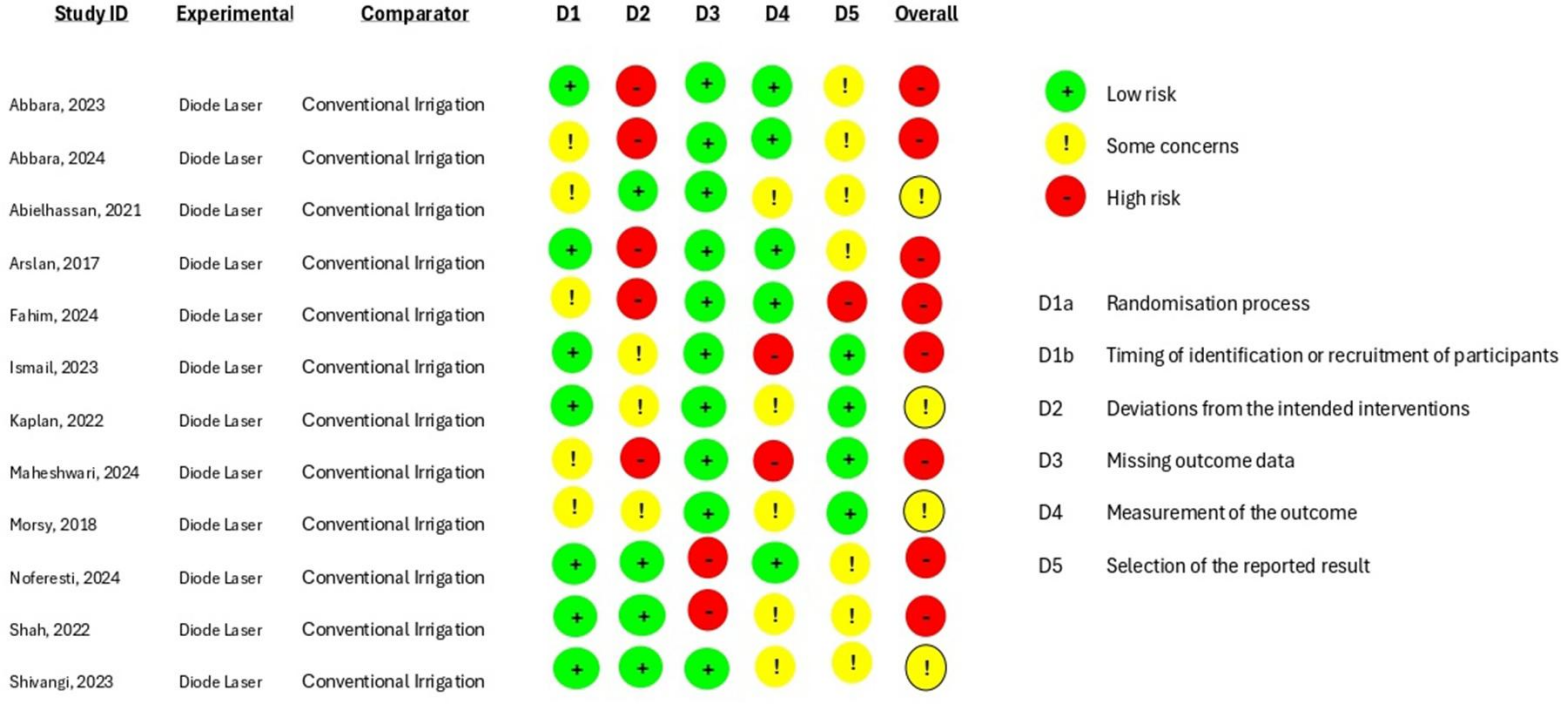
Risk of bias assessment (RoB 2.0) of the included randomized controlled trials.

The risk of bias assessment of the included randomized controlled trials, evaluated using the Cochrane Risk of Bias 2 (RoB 2) tool, is summarized in Figure 2. Overall, the methodological quality of the included studies was variable, with a predominance of studies classified as having high risk of bias or some concerns.

In the domain of the randomization process (D1), several studies demonstrated adequate methods and were judged as low risk; however, others raised concerns due to insufficient reporting or potential imbalance at baseline. Deviations from intended interventions (D2) were generally assessed as low risk in most studies, suggesting appropriate adherence to assigned treatments. Missing outcome data (D3) was also largely considered low risk, with most studies reporting complete or adequately addressed follow-up data.

In contrast, the measurement of outcomes (D4) showed greater variability, with some studies presenting high risk of bias, potentially related to lack of blinding or subjective outcome assessment, particularly for pain evaluation. Similarly, the selection of the reported results (D5) frequently raised concerns or was judged at high risk, reflecting possible selective reporting or insufficient protocol transparency.

As a result, the overall risk of bias was rated as high in several studies, with only a limited number classified as having low risk. These findings highlight important methodological limitations within the current evidence base and support cautious interpretation of the pooled results.

### Effects of diode Laser on outcomes

**Figure 3:**
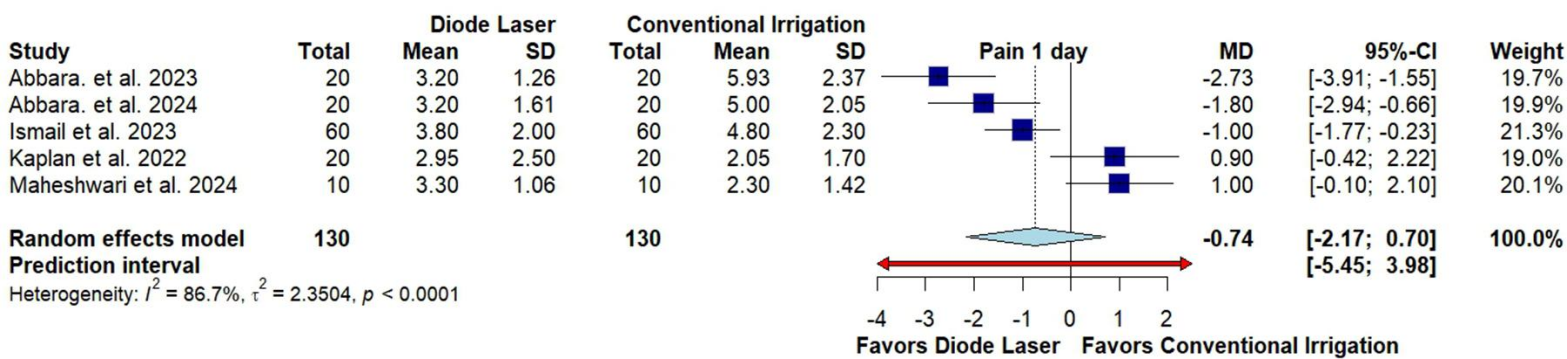
Forest plot of bacterial reduction for diode laser-assisted irrigation vs. conventional irrigation.

**Figure 4:**
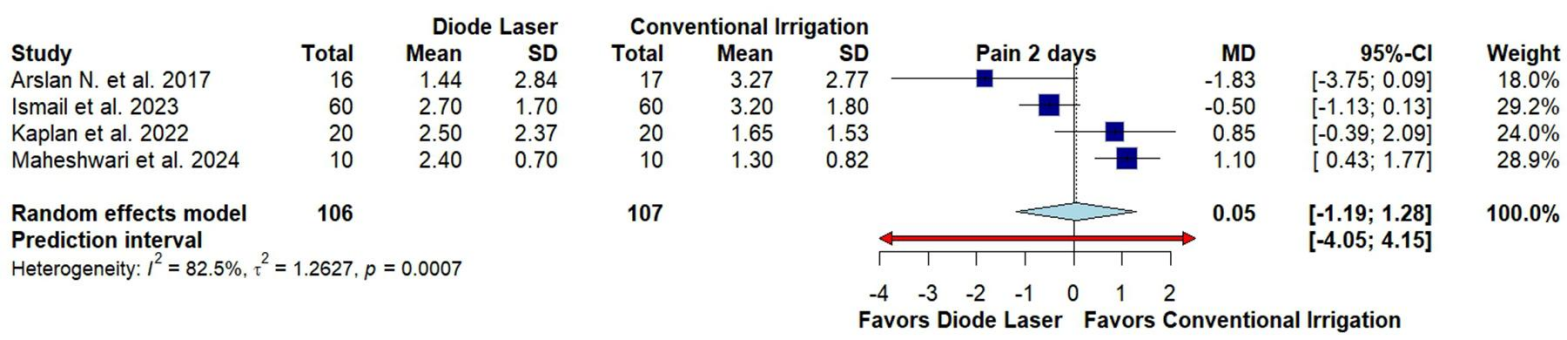
Forest plot of postoperative pain outcomes.

**Figure 5:**
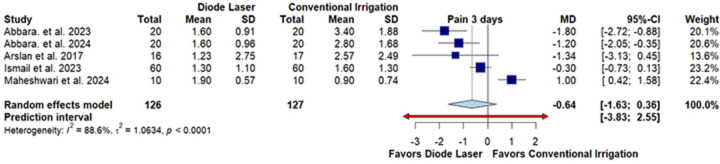
Forest plot of radiographic healing outcomes.

**Figure 6:**
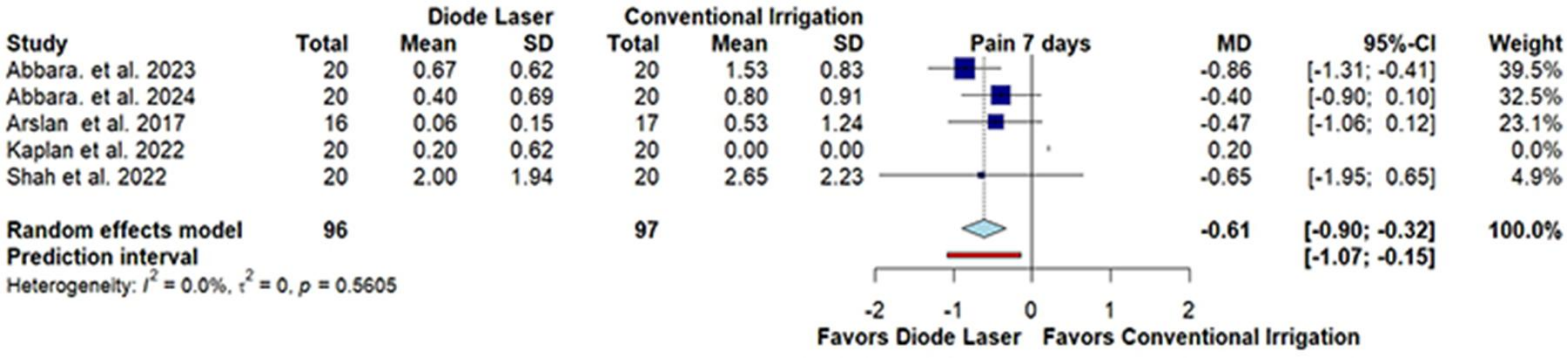
Forest plot of secondary outcomes (adverse events and procedure-related parameters).

**Figure 7:**
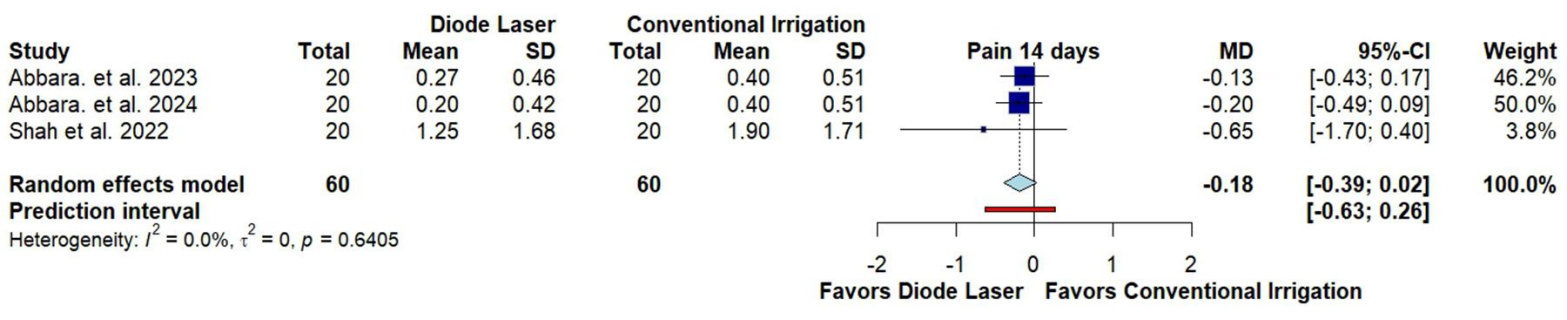
Subgroup/sensitivity analyses.

Postoperative pain outcomes were evaluated across multiple time points, as summarized in Figures 3–7. At 1 day post-treatment, the pooled analysis demonstrated a non-significant reduction in pain favoring diode laser compared to conventional irrigation (MD = −0.74; 95% CI: −2.17 to 0.70), accompanied by substantial heterogeneity (I^2^ = 86.7%) (Figure 3). Individual study estimates at this time point showed considerable variability, with some studies reporting a reduction in pain with diode laser, while others indicated minimal or no difference between treatment groups. At 2 days, the pooled estimate similarly indicated no statistically significant difference between groups (MD = 0.05; 95% CI: −1.19 to 1.28), with persistently high heterogeneity (I^2^ = 82.5%) (Figure 4), suggesting continued inconsistency in early postoperative outcomes. A comparable pattern was observed at 3 days, where diode laser demonstrated a trend toward reduced pain; however, this effect did not reach statistical significance (MD = −0.64; 95% CI: −1.63 to 0.36), and heterogeneity remained considerable (I^2^ = 88.6%) (Figure 5). These findings indicate that early postoperative pain responses varied substantially across studies, likely reflecting differences in study populations, intervention protocols, and outcome measurement methods. In contrast, at 7 days, the pooled analysis revealed a statistically significant reduction in pain associated with diode laser compared to conventional irrigation (MD = −0.61; 95% CI: −0.90 to −0.32), with no observed heterogeneity (I^2^ = 0.0%) (Figure 6), suggesting a more consistent effect at this later time point. By 14 days, no statistically significant difference between groups was observed (MD = −0.18; 95% CI: −0.39 to 0.02), and heterogeneity remained absent (I^2^ = 0.0%) (Figure 7), indicating convergence of pain levels between treatment modalities over time. Overall, while early postoperative outcomes were characterized by substantial heterogeneity and inconsistent findings, a more uniform reduction in pain favoring diode laser was observed at 7 days, followed by a lack of difference between groups at later follow-up.

**Figure 8:**
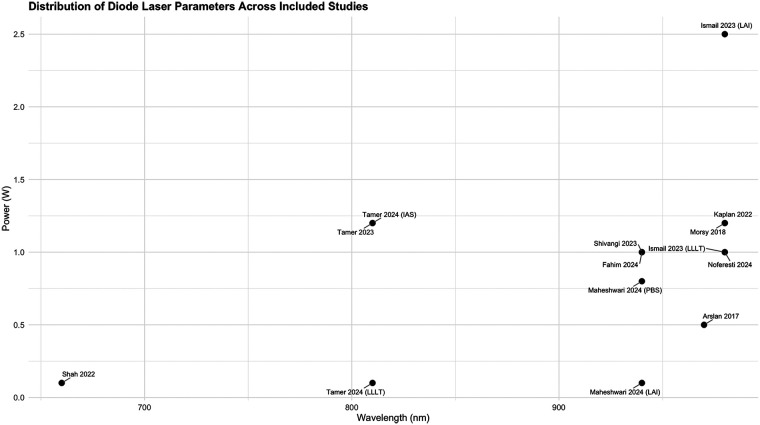
Funnel plot for publication bias assessment.

Figure 8 illustrates the distribution of diode laser parameters across the included randomized controlled trials, highlighting substantial variability in both wavelength and power settings. The wavelengths ranged from 660 nm to 980 nm, with most studies clustering between 940 nm and 980 nm.

Power output demonstrated a wide dispersion, ranging from low-power settings (0.1 W), typically used for photobiomodulation, to higher values up to 2.5 W applied for intracanal disinfection and irrigation activation. Notably, several studies reported similar wavelengths but markedly different power settings, reflecting heterogeneity in clinical protocols.

This variability underscores the lack of standardized laser parameters across studies and may contribute to differences in reported clinical outcomes.

## Discussion

This systematic review provides an updated synthesis of randomized controlled trials evaluating diode laser therapy as an adjunct to conventional irrigation in apical periodontitis. While previous reviews have reported comparable findings, the present study expands the evidence base by incorporating more recent trials and by evaluating a broader set of clinically relevant outcomes, including antimicrobial efficacy, postoperative pain, radiographic healing, and safety. In addition, this review systematically examines the heterogeneity of laser parameters across studies, which may partially explain the variability in reported outcomes. A key strength of this review lies in its rigorous methodological approach, including adherence to PRISMA 2020 guidelines, systematic study selection, and comprehensive risk of bias assessment.

Apical periodontitis is a challenging dental condition characterized by inflammation and infection of the periradicular tissues, often due to bacterial biofilms in the root canal system (35, 36). Effective management requires strategies to eradicate these biofilms, which are resistant to conventional treatments (37). Irrigant activation systems are crucial for optimizing the penetration of disinfecting agents, particularly in the apical third, enhancing the removal of pathogens and necrotic debris (26). The use of diode lasers in endodontic treatments is effective in reducing postoperative pain and improving root canal disinfection compared to conventional methods and mechanical irrigant activation systems (10, 38, 39). Furthermore, previous studies (40, 41) have highlighted the ability of diode lasers to penetrate up to 500 µm into dentinal tubules, effectively eliminating resistant bacteria such as *Enterococcus faecalis* and promoting better periapical tissue recovery. These findings underscore the potential of diode lasers to overcome the limitations of traditional methods, positioning them as valuable tools in the comprehensive management of apical periodontitis.

### Irrigation protocols and methodologies

The included studies demonstrated a variety of irrigation protocols, with sodium hypochlorite (NaOCl) and ethylenediaminetetraacetic acid (EDTA) serving as cornerstone irrigants. Advanced activation techniques, such as diode lasers with or without Er,Cr:YSGG, photodynamic therapy (PDT), and devices like the XP-Endo Finisher, were frequently employed in the experimental groups, whereas conventional protocols were followed in the control groups. This variation highlights the innovation in enhancing irrigant penetration and antimicrobial efficacy. However, the heterogeneity in adjuncts complicates direct comparisons between studies. Although diode lasers and complementary technologies appear promising, the inconsistency in activation protocols necessitates standardization to assess their clinical utility reliably.

The efficacy of irrigation protocols in endodontic treatment relies heavily on their ability to navigate the complex anatomy of the root canal system and effectively disinfect hard-to-reach areas. Sodium hypochlorite (NaOCl) and ethylenediaminetetraacetic acid (EDTA) remain fundamental irrigants due to their proven antimicrobial properties and ability to remove smear layers. However, advancements in irrigation activation methodologies, such as diode laser-activated irrigation (LAI) and sonic systems like EDDY, have further enhanced their clinical utility. It has been demonstrated that diode lasers significantly enhance antibacterial efficacy through thermophotodisruptive effects; however, their impact on postoperative pain reduction (PP) remains inconclusive, as no statistically significant differences were found between the conventional and advanced protocol groups (27). Similarly, LAI showed superior pain reduction compared to photobiostimulation and conventional irrigation within the first 24 h, although differences were not statistically significant beyond this period (28).

These findings underscore the need for standardizing laser parameters, as variability in settings such as wavelength, power, and activation duration complicates direct comparisons. For instance, diode lasers, with their deeper penetration into dentinal tubules, exhibit superior bacterial reduction (28), yet caution has been raised about inconsistent results linked to extruded debris from laser activation (27). The potential for advanced activation systems, such as LAI, to enhance irrigant penetration and reduce periapical inflammation positions them as promising adjuncts in treating apical periodontitis. However, their integration into routine protocols warrants further large-scale studies to elucidate their efficacy, particularly in addressing PP.

### Clinical outcomes

Primary outcomes, particularly postoperative pain and reduction in bacterial load, were consistently prioritized across the studies. Studies evaluating bacterial reduction relied on microbiological cultures, demonstrating the significant antimicrobial potential of diode lasers. Morsy et al. (10) reported statistically significant decreases in both aerobic and anaerobic bacterial counts following the application of a 980 nm diode laser, supporting its role as an effective adjunct to conventional endodontic treatment. Similarly, Wenzler et al. (2021) highlighted that the combination of diode laser irradiation (445 nm) with sodium hypochlorite irrigation resulted in a 92.7% reduction in bacterial load, surpassing the effectiveness of sodium hypochlorite alone.

Pain reduction, as measured by the Visual Analog Scale (VAS), yielded mixed results across studies. Arslan et al. (16) investigated the impact of low-level laser therapy (LLLT) on postoperative pain following root canal retreatment, reporting significant pain reduction within the first four days postoperatively compared to placebo. However, the difference was not statistically significant beyond this period​. In contrast, Morsy et al. (10) found that patients treated with diode laser therapy experienced significantly lower pain levels at all time intervals postoperatively, reinforcing its potential role in pain management.

Postoperative pain remains a pivotal concern in endodontics, often attributed to microbial and chemical factors, as well as debris extrusion during root canal procedures. Emerging evidence suggests that advanced laser-based techniques play a crucial role in improving pain management outcomes. Studies reported a significant reduction in pain scores when laser-assisted instrumentation was employed, particularly within the first 24 h, highlighting its role in mitigating inflammation associated with apical periodontitis (29). Similarly, other studies (30) have demonstrated that photodynamic therapy (PDT) significantly reduces pain at both 24 and 72 h by enhancing bacterial elimination and promoting tissue healing. Furthermore, the superiority of low-level laser therapy (LLLT) over conventional irrigation in managing immediate postoperative discomfort, particularly during the critical 24-hour period, has been emphasized (31).

Furthermore, radiographic healing, a crucial indicator of long-term treatment success, was underreported. Among the few studies that assessed periapical healing, Noferesti et al. (15) examined the impact of diode laser therapy in combination with calcium hydroxide (Ca(OH)₂) on periapical lesion resolution. Their findings revealed a reduction in periapical lesion scores at three and six months; however, the differences between the diode laser and Ca(OH)₂ groups were not statistically significant. These results suggest that while diode lasers may enhance healing, their long-term regenerative potential remains uncertain and requires further investigation.

Despite these promising results, it has been noted that while photon-induced photoacoustic streaming (PIPS) effectively disinfected root canals, its impact on postoperative pain was not significantly different from traditional irrigation techniques (42). In contrast, the combined application of diode lasers as both IAS and LLLT has been identified as the most effective protocol for sustained pain relief over a two-week period, particularly in cases of large apical lesions (32).

The elimination of microbial load within the root canal system is pivotal for the successful treatment of apical periodontitis. Advanced irrigation techniques, particularly those employing laser technology, have demonstrated superior bactericidal capabilities compared to conventional methods. It has been reported that photon-induced photoacoustic streaming (PIPS) with an Er: YAG laser significantly reduces bacterial levels in the apical third compared to conventional needle irrigation (CNI), demonstrating its efficacy in addressing biofilm complexities in the apical region (43). Similarly, the role of the SWEEPS mode of Er: YAG lasers in achieving effective bacterial reduction has been emphasized. However, its outcomes were comparable to those of passive ultrasonic irrigation (PUI) for specific bacterial targets (44). The combination of laser systems has also yielded notable results, as a dual laser protocol using Er, Cr: YSGG and diode lasers outperformed conventional sodium hypochlorite (NaOCl) and EDTA irrigation in reducing both aerobic and anaerobic bacterial counts (33). This highlights the enhanced ability of lasers to penetrate deep into dentinal tubules and eliminate resistant bacterial species, such as Enterococcus faecalis. These findings were corroborated by studies demonstrating the bactericidal effects of PIPS, which were particularly pronounced against facultative anaerobes, such as *Streptococcus* spp. This technique achieved effective disinfection in the root canal system, including areas inaccessible to traditional irrigation (45). However, it also underscored the potential for debris extrusion with laser systems, necessitating cautious application to minimize periapical inflammation.

The use of diode lasers in endodontics has gained attention for its potential to enhance bone regeneration through effective disinfection and stimulation of biological processes. A study evaluated single-visit regenerative endodontic procedures using low-power diode lasers compared to conventional irrigation and nano-chitosan solutions. The findings revealed that diode laser disinfection achieved a 67.7% success rate in restoring tooth sensibility and promoted significant periapical healing, although nano-chitosan solutions slightly outperformed lasers in terms of sensitivity recovery (34). Similarly, it was demonstrated that diode lasers, when combined with regenerative protocols such as platelet-rich fibrin (PRF) application, enhanced periapical bone healing and reduced lesion volume over 12 months. These findings underscore the role of diode lasers not only in microbial eradication but also in stimulating osteogenic activity, a critical factor in bone regeneration (46)

### Methodological strengths and limitations

The majority of studies employed randomized clinical trial designs with double-blind methodologies, enhancing internal validity. Randomization methods, such as computer-generated sequences and web-based tools, ensured allocation concealment. However, variations in follow-up durations, ranging from 72 h to six months, highlight inconsistencies in assessing long-term outcomes. Moreover, key variables such as periapical lesion size and secondary outcomes were frequently unreported, reflecting gaps in the comprehensive evaluation of treatment efficacy. Additionally, the demographic characteristics of study populations, including age ranges and sex distribution, were inconsistently detailed, further limiting the generalizability of findings.

Additionally, the overall risk of bias across included studies and the heterogeneity of intervention protocols limit the strength of quantitative conclusions. These factors underscore the need for cautious interpretation of pooled estimates and support the use of narrative synthesis as the primary basis for clinical inference.

Despite these strengths, the overall certainty of evidence remains limited due to heterogeneity in intervention protocols and risk of bias across included studies.

### Safety and complications

The studies uniformly reported an absence of significant postoperative complications, reinforcing the safety of diode lasers in endodontic practice. This finding is crucial, as patient safety remains a primary consideration when integrating new technologies into clinical workflows. Nevertheless, the lack of detailed reporting on potential minor adverse effects, such as thermal damage or tooth discoloration, suggests the need for more rigorous safety evaluations in future studies.

The application of diode lasers in endodontic treatment has demonstrated a favorable safety profile, with minimal reported complications when used within standardized protocols. The non-invasive nature of low-level laser therapy (LLLT) at 660 nm was highlighted, as it effectively reduces postoperative pain and inflammation without causing tissue overheating or structural damage during a 9-month follow-up (47). These findings underscore the laser's biostimulatory effects, which enhance healing and modulate pain without adverse outcomes. However, limitations such as small sample sizes and irregular follow-ups emphasize the need for larger trials to assess rare or delayed complications. These findings were corroborated, demonstrating that diode lasers, whether applied as an irrigation activation system (IAS) or through low-level laser therapy (LLLT), were well-tolerated across various patient groups (32). Their results identified no significant side effects, although the potential for localized mucosal irritation due to misaligned application was noted. This risk highlights the importance of operator training and adherence to rigorous procedural protocols. Despite the overall safety, potential hazards such as apical extrusion of irrigants during laser activation were identified. While lasers enhance irrigant penetration and biofilm removal, they may inadvertently push debris or solutions beyond the apex, increasing the risk of periapical inflammation. The critical importance of precise operator technique and calibrated laser parameters in mitigating these risks has been emphasized in various studies (32, 47).

Moreover, the combination of IAS and LLLT in diode laser applications provided superior outcomes in reducing postoperative pain compared to standalone techniques. This dual approach leverages both the disinfectant and biostimulatory properties of the diode laser, offering enhanced safety and efficacy. However, as highlighted by Shah et al., long-term follow-up and comparative studies are essential to validate these findings further.

### Implications for clinical practice and research

The results of this systematic review indicate that diode lasers hold promise as an adjunctive tool for improving antimicrobial efficacy and potentially reducing postoperative discomfort in endodontic treatments. However, the variability in outcomes and methodological inconsistencies necessitate cautious interpretation. Future studies should focus on standardized protocols, including uniform laser settings, treatment durations, and follow-up intervals, to enable meaningful comparisons. Moreover, integrating advanced imaging techniques, such as cone-beam computed tomography (CBCT), could provide more precise assessments of periapical healing and bone regeneration.

The integration of diode lasers into endodontic protocols represents a significant advancement in achieving superior treatment outcomes. Their proven ability to enhance disinfection, reduce postoperative pain, and potentially promote healing has positioned diode lasers as a valuable adjunct in managing complex endodontic cases.

### Clinical practice

Diode lasers, particularly when used in low-level laser therapy (LLLT) or laser-activated irrigation (LAI), have consistently demonstrated efficacy in reducing postoperative pain. Significant reductions in pain scores within the first 48 h post-treatment have been reported, emphasizing the anti-inflammatory effects of diode lasers on periapical tissues (29–31). These findings support the routine use of these devices in symptomatic apical periodontitis cases, where patient comfort is a primary concern. Furthermore, the enhanced disinfection achieved by diode lasers, through their ability to penetrate dentinal tubules and eradicate resistant species such as *Enterococcus faecalis*, provides clinicians with a reliable method to ensure more predictable healing outcomes.

While safety profiles remain favorable, with minimal reported complications such as mucosal irritation or apical extrusion of irrigants, proper operator training and adherence to standardized protocols are critical. This includes carefully calibrating power settings and applying controlled techniques to prevent tissue damage and optimize outcomes. The cost-effectiveness and portability of diode lasers also make them an accessible option for a wide range of clinical settings, further supporting their broader adoption.

Despite the promising results, several gaps remain that warrant further investigation. Standardization of laser parameters, including wavelength, pulse duration, and power, is essential to ensure consistent and reproducible clinical outcomes. Current studies emphasize the variability in methodologies, highlighting the need for standardization to ensure consistent and reproducible outcomes (30), which limits direct comparisons across trials. Long-term studies are crucial to understanding the impact of diode lasers on periapical healing and the prevention of recurrent infections. While immediate postoperative pain relief and microbial reduction have been well-documented, the role of diode lasers in sustained bone regeneration and tissue healing requires further exploration. Comparative trials with other laser systems, such as Er: YAG and photodynamic therapy (PDT), could provide valuable insights into the unique advantages and limitations of diode lasers. Patient-centric research focusing on quality-of-life metrics, such as comfort, satisfaction, and functional recovery, would also enhance the clinical relevance of future studies. Additionally, the potential of diode lasers in managing cases with extensive apical lesions or challenging anatomies offers an exciting avenue for research.

An important clinical implication emerging from this review is the potential role of diode laser therapy as an adjunctive or alternative approach in selected scenarios. Although current evidence suggests that laser-assisted irrigation achieves comparable effects to conventional methods rather than clear superiority, it may offer practical advantages in specific patient populations. For instance, in cases where patients present intolerance, hypersensitivity, or limited response to conventional irrigants, diode laser therapy could represent a complementary strategy. However, this perspective remains hypothesis-generating and should be interpreted cautiously, given the limited strength and heterogeneity of the available evidence.

## Conclusion

Diode laser therapy appears to provide comparable clinical outcomes to conventional irrigation methods when used as an adjunct in the management of apical periodontitis. However, due to heterogeneity in study protocols and overall risk of bias, current evidence remains insufficient to support its routine use as a superior alternative. Future well-designed randomized trials with standardized laser parameters are required to clarify its clinical role.

## Data Availability

The original contributions presented in the study are included in the article/Supplementary Material, further inquiries can be directed to the corresponding author/s.
