# Supplementary material for: Efficacy of diode Laser compared to conventional irrigation in endodontic treatment of apical periodontitis: a systematic review and meta-analysis
Source: Front Oral Health. 2026 May 29;7:1814056. doi: 10.3389/froh.2026.1814056 (PMC13260606; doi:10.3389/froh.2026.1814056)
Supplement: Supplementary file 1 [file Table1.docx]

**Table 1. Characteristics of the included studies**

| **First author** | **Year** | **Country of study** | **Study design** | **Sample size** | **Randomization method** | **Blinding** | **Duration of follow-up** | **Number of patients included in each group** | **Age of patients (mean, range)** | **Sex of patients** | **Inclusion criteria** | **Exclusion criteria** |
| --- | --- | --- | --- | --- | --- | --- | --- | --- | --- | --- | --- | --- |
| Abbara. et al. | 2023 | Syria | Randomized clinical trial, double-blind | 60 | Random allocation with www.random.org | Double-blind (patient and evaluator) | 14 days | 20 | 25 to 44 years | 32 men, 28 women | Maxillary incisors with asymptomatic apical periodontitis and apical lesion > 5 mm | Patients with systemic diseases, pregnant, non-restorable teeth |
| Abbara. et al. | 2024 | Siria | randomized clinical trial, single-blind | 80 | Randomization via software (random.org) | Single-blind (patient) | 14 days | 20 | 32.61 years (25 to 44 years) | 45% men, 55% women | Maxillary incisors with asymptomatic necrosis and large periapical lesions | Systemic diseases, non-restorable teeth, sensitivity to lidocaine, advanced periodontitis |
| Abielhassan et al | 2021 | Egipto | randomized clinical trial, double-blind | 45 | Random allocation via folded paper numbers | Double-blind | 6 and 12 months | 15 | 18 to 50 years | Men and women, unspecified distribution | Mature anterior teeth, pulp necrosis, periapical lesion | Non-restorable teeth, root resorptions, drug sensitivity |
| Arslan N. et al. | 2017 | Turkey | Triple-blind, randomized controlled trial | 36 | Computer-generated randomization (www.randomizer.org) | Triple-blind | 7 days | 18 | Average of 29 years | Low-level laser therapy: 8Men / 10woman, Placebo: 13Men / 5women | Mandibular molars requiring RCR, periapical lesion (PAI 2-3), preoperative pain VAS <50, percussion pain VAS <50 | Periodontal probing >3mm, swelling, sinus tract, palpation sensitivity |
| Fahim et al. | 2024 | Egypt | Randomized clinical trial, double-blind | 30 | Random allocation with www.randomizer.org | Double-blind (patient and microbiologist) | Not specified for long-term follow-up | 10 | 18 to 35 years | Not specified | Anterior single-rooted teeth, pulp necrosis and asymptomatic apical periodontitis | Recent antibiotic use, systemic diseases, allergy to NSAIDs |
| Ismail et al. | 2023 | Egypt | Randomized clinical trial, single-blind | 180 | Random allocation via sealed envelopes | Single-blind (patient) | 72 hours | 60 | 18 to 50 years | Only men | Patients with symptomatic apical periodontitis in mandibular molars | Systemic diseases, prior use of analgesics |
| Kaplan et al. | 2022 | Turkey | Randomized clinical trial, single-blind | 80 | Random allocation via random.org | Single-blind (patient) | 7 days | 20 | 18 to 65 years | 40% men, 60% women | Mandibular molars with symptomatic apical periodontitis | Pregnancy, recent use of analgesics or antibiotics, teeth with trauma or non-restorable |
| Maheshwari et al. | 2024 | India | Randomized clinical trial, in vivo | 30 | Not specified | Not mentioned | 72 hours | 10 | Not specified | Not specified | Molars with symptomatic apical periodontitis, untreated, without inflammatory exudate | Teeth with open apices, systemic diseases, recent analgesic use |
| Morsy et al | 2018 | Egypt | Randomized controlled trial | 56 | Computer-generated sequence, opaque sealed envelopes | Double-blind for pain and microbiological evaluation; single-blind for periapical lesion size | 7 days | 28 | 18-35 years | Diode laser group: 14Men/14women, Control group: 10Men/18women | Adults 18-35 years, necrotic pulp in maxillary central incisors, closed apex, periapical radiolucency, no systemic disorders, no recent antibiotics or analgesics. | Pregnant women, systemic disorders, open apex, previous endodontic treatment, mobility > Grade I, periodontal pocket >4mm. |
| Noferesti et al | 2024 | Iran | Randomized controlled clinical trial | 19 | Sealed envelope allocation | Triple-blind | 3 months and 6 months | 12 | 18-50 years | Ca(OH)2 + Laser Irrigation 25% Men / 75% Women, Ca(OH)2: 36% Men / 66% Women | Single-rooted teeth with previous RCT, age 18-50, periapical lesion present, no significant medical conditions | Pregnancy, recent antibiotic use, hospitalization history, mobile teeth, open apex, severe periodontal disease |
| Shah et al. | 2022 | India | Randomized controlled trial, double-blind | 40 | Random allocation via sealed envelopes | Double-blind (patient and investigator) | 9 months | 20 | Not specified | Not specified | Single-rooted teeth with non-vital pulps and apical periodontitis | Multi-rooted teeth, systemic conditions, dental deformities |
| Shivangi et al. | 2023 | India | Double-blind randomized controlled trial | 90 | Simple randomization (manually generated sealed envelopes) | Double-blind (patients and evaluator) | 72 hours | 30 | 18-60 years | Not specified | Patients aged 18–60, single-rooted teeth, pulp necrosis, symptomatic apical periodontitis | Uncontrolled systemic diseases, previous root canal treatment, severe periodontal disease |

**Table 2. Characteristics of outcomes**

| First author | Year of publication | Irrigation protocol | Primary outcome | Secondary outcomes | Measurement method | Time points for outcome measurement |
| --- | --- | --- | --- | --- | --- | --- |
| Zakaria S. et al. | 2024 | **1.- Control Group - Conventional Irrigation (NaOCl/EDTA)** Primary irrigation: 5 ml of 2.5% NaOCl after each file instrumentation. Applied using a 30G side-vented needle, 2 mm short of working length. Intermediate rinse: 5 ml of sterile saline. NaOCl neutralization: 1 ml of 5% sodium thiosulfate. Final irrigation: 5 ml of 17% EDTA for 1 minute to remove the smear layer. **2 . Intervention Group 1 - Dual Laser (Er,Cr:YSGG + Diode 940 nm) Er,Cr:YSGG laser for smear layer removal and Diode laser (940 nm) for bacterial disinfection enhances root canal decontamination.** Irrigation Protocol Initial rinse: 5 ml of sterile saline. Er,Cr:YSGG laser application: Wavelength: 2780 nm. Power: 1.25 W. Frequency: 20 Hz. Mode: Pulsed. Air/Water: 10% air, 1% water. Optical fiber: 200 µm radial RFT2 fiber. Application method: 4 cycles of 10 seconds, with 10-second pauses. Function: Smear layer removal. Intermediate rinse: 5 ml of sterile saline. Diode laser (940 nm) application: Wavelength: 940 nm. Power: 1 W (continuous mode). Optical fiber: 200 µm flexible fiber. Applied in a helical motion, 1 mm short of working length. Application method: 4 cycles of 10 seconds, with 10-second pauses. Function: Bacteria elimination up to 1000 µm into dentin. **3. Intervention Group 2 - EDTA + Diode Laser 940 nm** Evaluate whether combining EDTA for smear layer removal and Diode laser (940 nm) for bacterial disinfection is a cost-effective alternative to the dual laser protocol. Irrigation Protocol: Initial irrigation: 5 ml of 17% EDTA for smear layer removal. Intermediate rinse: 5 ml of sterile saline. Diode laser (940 nm) application: Wavelength: 940 nm. Power: 1 W (continuous mode). Optical fiber: 200 µm flexible fiber. | **Control Group (NaOCl/EDTA):** Total bacterial reduction: 57.6%. Showed the lowest reduction in bacterial load. **Dual Laser Group (Er,Cr:YSGG + Diode 940 nm):** Total bacterial reduction: 99.97%. Achieved the highest bacterial elimination. **Combined Group (EDTA + Diode 940 nm):** Total bacterial reduction: 99.94%. Comparable to the dual laser group. **Primary Outcome Interpretation** Both laser-assisted protocols (Dual Laser and EDTA + Diode) were significantly superior in bacterial reduction compared to the conventional NaOCl/EDTA irrigation protocol. No significant difference between the Dual Laser and the EDTA + Diode Laser groups, indicating that EDTA + Diode laser may be a more cost-effective alternative to the Er,Cr:YSGG + Diode combination. | Not specified | CFU count in aerobic and anaerobic cultures | Before and after intervention |
| Tamer M. et al. | 2023 | **1.- Control Group 1- Passive Ultrasonic Irrigation (PUI)** A #25 U-file ultrasonic tip (Zipperer Co.) was mounted on a Woodpecker ultrasonic handpiece. Inserted 2 mm short of the apex without contacting the canal walls.NaOCl (5.25%) was activated for 45 seconds at 30 kHz (medium power setting) using a push-pull motion. 2 mL of irrigant per cycle, repeated until 40 mL of NaOCl was used (~15 min total). Final Irrigation: EDTA 17% (activated for 15 sec, repeated twice). CHX 2% (activated for 15 sec, repeated twice). **2.- Control Group 2** - **XP-Endo Finisher File Activation** Irrigation Activation Protocol: The XP-Endo Finisher file (#25, non-tapered, NiTi alloy) was inserted 2 mm short of the apex. Operated at 800 rpm, 1 N·cm torque. Moved along the entire canal perimeter to enhance debris removal. 2 mL of NaOCl(5.25%) per cycle, repeated until 40 mL total (~15 min). Final Irrigation: EDTA 17% (activated for 15 sec, repeated twice).CHX 2% (activated for 15 sec, repeated twice). **3.- Intervention Group 3 - Diode Laser (810 nm) Activation** Irrigation Activation Protocol: A 200 µm optical fiber tip (Mercury G10, Wuhan Pioon Technology) was inserted 2 mm short of the apex. Operated at: 1.2 W (average power). 50 Hz frequency. 12 J per cycle (pulsed mode). The fiber tip was moved apico-coronally in a slow helical motion. NaOCl (5.25%) was activated for 10 sec per cycle (10 sec pause). 2 mL per cycle, repeated until 40 mL total (~15 min). Final Irrigation: EDTA 17% (activated for 15 sec, repeated twice). CHX 2% (activated for 15 sec, repeated twice). | **Postoperative pain (VAS)** : **The Diode Laser (810 nm) group had the lowest postoperative pain across all time points.** Day 1 pain (3.20 ± 1.26) was significantly lower than both the PUI group (5.00 ± 2.14) and the XP-Endo group (5.93 ± 2.37) (p = 0.002). Day 3 pain was also lower in the Diode Laser group (1.60 ± 0.91) compared to PUI (2.80 ± 1.86) and XP-Endo (3.40 ± 1.88) (p = 0.017). **The XP-Endo Finisher file group had the highest postoperative pain levels:** Day 1 pain was the highest (5.93 ± 2.37), suggesting greater mechanical disruption and extrusion of debris. Pain levels remained significantly elevated even at Week 1 (1.53 ± 0.83) compared to the Diode Laser group (0.67 ± 0.62). **Passive Ultrasonic Irrigation (PUI) showed moderate pain levels:** Pain was lower than XP-Endo but higher than the Diode Laser group. Pain reduction was gradual over time but was significantly lower than XP-Endo at Week 1 (p = 0.006).**All groups experienced complete pain resolution by Week 2.** | Not specified | VAS scale(mean +-sd) | 1, 3, 7, and 14 days |
| Santos M. et al. | 2019 | **1. Control Group (CG) – Standard Root Canal Treatment without PDT** Irrigation protocol: 40 mL of 2.5% NaOCl (sodium hypochlorite). Passive ultrasonic irrigation (PUI) using a 20.01 ultrasonic tip (Irrigonic, Helse Ultrasonics, Brazil) for 3 cycles of 20 seconds. Final rinse with 17% EDTA (3 minutes). **2. Intervention Group: Photodynamic Therapy (PDT)** – Root Canal Treatment with PDT. Same protocol as the Control Group, with the addition of PDT after instrumentation: PDT protocol: Methylene blue (MB) at 1.56 μM/mL was applied inside the canal for 2 minutes. A 660 nm diode laser (Laser Duo, MMOptics, Brazil) was used. A 25.04 optical fiber was inserted to the working length (WL) and moved vertically in a gentle motion. Laser settings: Power: 100 mW. Energy density: 600 J/cm². Total energy applied: 18 J. Laser was applied for 3 minutes. Methylene blue was removed with saline irrigation. | Postoperative pain (VAS) | Not specified | VAS scale at different time points (mean +-sd) | 24, 72 hours, and 1 week |
| Maheshwari P. et al. | 2024 | **1. Control Group** - Mock Laser (ML) / Placebo: Irrigation protocol: 2.5% NaOCl (2 mL per canal). Final rinse: 1.5 mL of 17% EDTA followed by 1.5 mL of saline. Mock Laser Procedure: A laser handpiece was placed near the treated tooth, but no actual laser emission was applied. **2. Intervention Group 1 - Photobiostimulation (PBS) in the Periapical Region** Root Canal Preparation Protocol: Same protocol as the control group (Mock Laser). Laser Therapy Protocol: Laser device: Epic X Biolase diode laser. Wavelength: 940 ± nm. Power: 0.8 W (continuous mode). Energy density: 70 J/cm². Application method: The laser tip was applied perpendicular to the buccal and lingual/palatal mucosa. Two sessions of 20 seconds each with a 5-second gap between sessions. The laser fiber was kept 10 mm away from the tissue to ensure non-invasive penetration. **3. Intervention Group 2 - Laser-Activated Irrigation (LAI) / Intracanal Laser Disinfection:** Root Canal Preparation Protocol: Same as the control group. Laser Therapy Protocol: Laser device: Epic X Biolase diode laser. Wavelength: 940 ± nm. Power: 0.1 W (continuous mode). Application method: The laser tip was inserted 1 mm short of the working length (WL). Moved from orifice to apex in a circular motion. Four cycles of activation per canal, each with 10-second intervals between cycles. | Postoperative pain (VAS) | Not specified | Visual Analog Scale (VAS) (mean+-sd) | 24, 48, and 72 hours |
| Kaplan T. et al. | 2022 | **1. Control Group 1 - Conventional Irrigation (CI)** Irrigation Protocol: 5.25% NaOCl (5 mL) between instruments. Final irrigation: 5 mL of 5.25% NaOCl (31G side-vented needle, 2 mm short of WL). 5 mL of 17% EDTA for 1 min (smear layer removal). 5 mL of saline to neutralize residues. Intracanal Medication: Calcium hydroxide (Ca(OH)₂) paste. Temporary Restoration: Sterile cotton + Cavit-G (3M ESPE, USA). **2. Group 2 - Sonic Irrigation Activation (EDDY):** Irrigation Activation with EDDY (VDW, Germany): Activation with a 25/04 polyamide tip at 6,000 Hz. Three cycles of 20-second NaOCl activation, 2 mm short of WL. EDTA activation for 30 seconds. Final rinse identical to Group 1. Intracanal Medication and Temporary Restoration: Same as Group. **3. Intervention Group 3 - Conventional Irrigation + Diode Laser Disinfection:** Root Canal Preparation and Conventional Irrigation: Same protocol as Group 1. Laser Disinfection Protocol: Laser Device: 980 nm diode laser (Medency Primo, Italy). Power: 1.2 W (pulsed mode, 50 Hz). Energy per cycle: 12 J per cycle. Application: 4 cycles of 10-second irradiation. Fiber tip (200 µm) positioned at WL. Slow helical movement (2 mm/s) from apical to coronal. Intracanal Medication and Temporary Restoration: Same as Group 1. **4. Intervention Group 4 - Sonic Irrigation Activation + Diode Laser Disinfection: Root Canal** Preparation and Sonic Activation: Same protocol as Group 2. Laser Disinfection Protocol: Same protocol as Group 3. Intracanal Medication and Temporary Restoration: Same as Group 1. | Postoperative pain (NRS) | Analgesic use | Numeric pain scale (NRS) | 8, 24, 48 hours, and 7 days |
| Ismail H. et al. | 2023 | **1. Intervention Group 1 – Laser-Activated Irrigation (LAI):** Irrigation Protocol: 2.5% NaOCl irrigation between files. Final rinse: 2 mL of 17% EDTA. 3 mL of normal saline. Diode Laser Irrigation Activation: Laser device: Master Lase/Expert Lase (KaVo, Germany). Wavelength: 980 nm. Power: 2.5 W. Frequency: 100 Hz. Application: Optical fiber introduced 2–3 mm short of working length. Apical-to-coronal withdrawal motion. Each canal irradiated for 20 seconds, repeated three times (total 60 sec). **2. Intervention Group 2 – Low-Level Laser Therapy (LLLT)** Root Canal Preparation: Same as Group 1. Diode Laser Therapy Protocol: Laser Device: Lite Medics (serial no. 148 Ver. SwvM. 150VS108VT.100). Wavelength: 980 nm. Power: 1.0 W (continuous mode). Frequency: 10 Hz. Application: Laser applied extra-orally (3 mm from oral mucosa). Beam focused on the apical region of the treated tooth. Laser exposure: 30 seconds on buccal + 30 seconds on lingual side. **3. Control Group 3 – Mock Laser (ML):** Root Canal Preparation & Irrigation: Same as Group 1. Mock Laser Procedure: Laser handpiece positioned near the treated tooth without activation (placebo). | Postoperative pain (VAS) | None | VAS at 24, 48, and 72 hours | 24, 48, and 72 hours |
| Shah D. et al. | 2021 | **1.- Intervention Group I – Root Canal Treatment (RCT) + Low-Level Laser Therapy (LLLT)**: Irrigation Protocol: 5% NaOCl (10 mL) (Vensons India) as the main irrigant. 17% EDTA (Vishal Dentocare Pvt Ltd) for smear layer removal. 2% chlorhexidine (CHX) (STEDMAN Pharmaceutical) as an antibacterial rinse. Intracanal Medicament: Aqueous 2% CHX mixed with calcium hydroxide (Ca(OH)₂) (DentPro, India). Placed using a #20 lentulo spiral (Dentsply Maillefer). Temporary restoration: Cavit-G (3M ESPE, Germany). Low-Level Laser Therapy (LLLT) Protocol: Laser Device: 660 nm Duolase (INDILASE, India). Power: 100 mW. Mode: Continuous wave. Application: Periapical region irradiated for 1 minute per session. Total of 3 sessions: Day 0, Day 7, and Day 14. **2.-Control Group II – Root Canal Treatment (RCT) Without LLLT: Root Canal Preparation and Irrigation:** Same protocol as Group I. Intracanal Medicament: Same as Group I (2% CHX + Ca(OH)₂). Mock Laser Therapy (Placebo Control): Laser device positioned but not activated. | Postoperative pain (VAS) | Reduction in periapical lesion size | VAS and digital radiographs | Preoperative, 3, 6, and 9 months |
| Abielhassan M. et al | 2021 | **1. Intervention Group A – Diode Laser Disinfection (Intervention 1):** Irrigation Protocol: 1.25% NaOCl (20 mL final flush). 17% EDTA. Diode Laser Disinfection Protocol:Laser device: SiroLaser Blue. Wavelength: 940 nm. Fiber tip insertion: 1 mm short of working length (WL). Circumferential laser beam application for 2 seconds per cycle. 10 interrupted cycles of laser irradiation (5-second gap per cycle). Total contact time: 20 seconds. Regenerative Protocol: Platelet-rich fibrin (PRF) scaffold was prepared by centrifuging the patient’s blood at 4,500 rpm for 12 minutes. PRF was packed inside the canal, followed by a collagen plug. Biodentine was applied as the coronal barrier. Final restoration: Resin-modified glass ionomer. **2. Intervention Group B – Nano Chitosan Disinfection (Intervention 2): Nano Chitosan Disinfection Protocol:** Solution: 0.2% nano chitosan irrigation. Delivery method: Side-perforated needle inserted 1 mm short of WL. Volume: 20 mL total. Application: 4 injections at 3-minute intervals. Regenerative Protocol: Same as Group A (PRF, collagen plug, Biodentine, final restoration). **3. Control Group C – Conventional Irrigation: Root Canal Preparation & Irrigation:** Same as Group A. Final irrigation: 1.25% NaOCl (20 mL). 17% EDTA (3 minutes). Regenerative Protocol: Same as Group A. | Survival of treated teeth | Dental sensitivity test | CBCT and sensitivity test with pulpotomy | 6 and 12 months |
| Tamer M et al. | 2024 | **1. Control Group 1 – No Laser Application:** Irrigation Protocol: 5.25% Sodium Hypochlorite (NaOCl) (40 mL total). 17% EDTA for 15 sec (repeated twice). 2% Chlorhexidine (CHX) for 15 sec (repeated twice). Irrigation Activation: Passive Ultrasonic Irrigation (PUI) using #25 U-file tip (30 kHz, push-pull movement). **2. Intervention Group 2 – Diode Laser 810 nm as an Irrigation Activation System (IAS):**  Root Canal Preparation & Irrigation: Same as Group 1. Diode Laser Irrigation Activation: Laser device: Mercury G10 (Wuhan Pioon Technology, China). Wavelength: 810 nm. Fiber tip insertion: 2 mm short of the apex. Settings: Peak Power: 2.4 W. Average Power: 1.2 W. Frequency: 50 Hz (pulsed mode, 5 ms). Total Energy: 12 J per cycle. Application: Slow helical movement apico-coronally. Each 2 mL of irrigant activated for 45 sec. Repeated until 40 mL of NaOCl was activated (15 min total). Final Irrigation Activation: EDTA and CHX were also activated with the diode laser for 15 sec (repeated twice). **3. Intervention Group 3 – Diode Laser 810 nm for Low-Level Laser Therapy (LLLT): Root Canal Preparation & Irrigation:** Same as Group 1. Low-Level Laser Therapy (LLLT) Protocol: Laser Device: Mercury G10 (Wuhan Pioon Technology, China). Wavelength: 810 nm. Power: 100 mW (continuous mode). Frequency: 10 Hz. Application: Laser tip positioned 3 mm away from the mucosa. Irradiation applied perpendicularly to the apical region. 80 sec per site: 80 sec on the buccal and 80 sec on the palatal. **4. Intervention Group 4 – Diode Laser 810 nm as IAS + LLLT (Combined):** Root Canal Preparation & Irrigation: Same as Group 2 (IAS). Diode Laser Activation & LLLT: Same as Group 3 (LLLT) after obturation. | Postoperative pain (VAS) | Not specified | VAS scale at 1, 3, 7, and 14 days | 1, 3, 7, and 14 days |
| Shivangi S. et al. | 2023 | **1. Control Group 1 – Continuous Rotary Instrumentation Without Laser: Instrumentation:** Endostar E3 Azure rotary files (heat-treated NiTi system). **Crown-down technique to apical size based on gutta-percha fit.**Irrigation Protocol: 5.25% Sodium Hypochlorite (NaOCl) (5 mL per canal). Manual dynamic agitation with fitted gutta-percha cone (100 strokes/min). Final rinse: 5.25% NaOCl (1 min). 17% EDTA (1 min). **2. Intervention Group 2 – Continuous Rotary Instrumentation with Laser Irradiation:** Root Canal Preparation & Irrigation: Same as Group 1. Diode Laser Therapy Protocol: Laser device: Biolase Epic X (Biolase Inc., California, USA). Wavelength: 940 nm. Fiber tip: 200 µm diameter. Power: 1 W (pulsed mode). Application: Laser tip positioned 2 mm short of working length (WL). Five-second irradiation cycles with 10-second pauses (4 cycles). Tip moved coronally in a helical motion. **3. Intervention Group 3 – Mechanized Reciprocating Handpiece** Instrumentation with Laser Irradiation: Same as Group 1. Instrumentation: K-Flex stainless steel files (Sybron Endo, USA) used with K-400 reciprocating handpiece (Bombay Dental, India). Oscillation: 30° forward, 30° backward at 3000 rpm. Step-back preparation technique. Diode Laser Therapy: Same as Group 2. | Postoperative pain reduction | Percussion pain reduction, need for rescue medication | Visual Analog Scale (VAS) | Baseline, 24, 48, and 72 hours |
| Morsy D. et al | 2018 | **1. Intervention Group – Diode Laser (DL) Desinfection:** Irrigation Protocol: 2.5% Sodium Hypochlorite (NaOCl) (10 mL between files). 17% EDTA (5 mL) for smear layer removal. Final rinse: 5 mL sterile saline. Diode Laser Disinfection Protocol: Laser device: Lite Medics (Italy). Wavelength: 980 nm. Fiber tip: 200 µm diameter. Power: 1.2 W (pulsed mode). Application: Laser tip positioned 1 mm short of working length. Irradiation cycle: 5 seconds irradiation, 10 seconds pause. Total of 4 cycles per tooth. Helical movement from apical to coronal portion. **2. Control Group – Conventional Endodontic Treatment (Placebo):** Root Canal Preparation & Irrigation: Same as Experimental Group. Placebo Procedure: Laser fiber optic placed inside the root canal but NOT activated. Temporary Restoration and Pain Measurement: Same as Experimental Group. Microbiological Analysis: Bacterial samples collected at S1-S5 (same protocol as the Experimental Group). | Post-Endodontic Pain (NRS Scale) | Bacterial Load Reduction (Colony Forming Units - CFU/mL) | Pain intensity was measured using the NRS scale from 0 to 10 | Preoperative (Baseline)  6 hours post-treatment  12 hours post-treatment  24 hours post-treatment  48 hours post-treatment  7 days post-treatment |
| Noferesti M. et al | 2024 | **1. Internvention Group – Calcium Hydroxide + Laser Irradiation (Ca(OH)₂ + LI):** Irrigation Protocol: 5.25% Sodium Hypochlorite (NaOCl) (1 mL per file change, passive delivery). Final rinse: 17% EDTA (5 min) + 5.25% NaOCl (1 min). Intracanal Medication: Calcium hydroxide paste (Gholchadent, Golchai, Iran) for 10 days.Temporary restoration with Coltosol (Coltene). Laser Irradiation Protocol (Second Appointment): Laser Device: Doctor Smile 980 nm diode laser. Fiber Tip: Non-initiated 200-μm fiber. Power Output: 1W (Continuous Wave - CW mode). Irradiation Protocol: Irradiation performed for 2 sec per mm of working length. Two irradiation cycles per canal (30 sec interval between cycles). Tip movement: Helical motion from apex to coronal. **2. Control Group – Calcium Hydroxide Only (Ca(OH)₂):** Root Canal Retreatment Protocol: Same as the Experimental Group. Intracanal Medication: Same as the Experimental Group (Ca(OH)₂ for 10 days). Laser Therapy: No laser was applied in this group. | Periapical Healing (PAI Score) | Clinical Success (Reduction in Symptoms) | **Outcome Primary:** Radiographic Assessment Method: Periapical Index (PAI) Score. **Outcome Secondary:** Pain Assessment Method: Visual Analog Scale (VAS) | **1. Time Points for Periapical Healing Measurement:** Baseline (Pre-treatment)  3 months post-retreatment  6 months post-retreatment **2. Time Points for Clinical Symptom Measurement:**  Pain (VAS Score: 0–10) ----- Baseline, 3 months, 6 months. Swelling (None, Mild, Moderate, Severe) ---Baseline, 3 months, 6 months. Tenderness (None, Mild, Moderate, Severe) --- Baseline, 3 months, 6 months |
| Arslan N. et al. | 2017 | **1. Intervention Group – LLLT After RCR:** Irrigation Protocol: 1% Sodium Hypochlorite (NaOCl) (2 mL per file change). Final rinse: 5 mL 1% NaOCl, 2 mL 2% citric acid (1 min), 5 mL distilled water.Intracanal Medication: Calcium hydroxide paste (Sultan Healthcare Inc., USA) for 7 days. Temporary restoration with Cavit-G (3M ESPE). Laser Irradiation Protocol: Laser Device: SIROLaser Xtend (Sirona Dental Systems, Germany). Wavelength: 970 ± 15 nm. Power Output: 0.5 W. Pulse Frequency: 10 Hz. Irradiation Protocol: Mesial and distal root apexes irradiated for 30 sec. Power density: 2.86 W/cm². 200-μm optical fiber and bleaching application tip used. Tip placed ~10 mm from the tissue. **2. Control Group – Placebo Laser After RCR:** Root Canal Retreatment Protocol: Same as the Experimental Group. Placebo (Mock) Laser Application: Laser tip placed similarly to the experimental group. Laser was NOT activated. | Postoperative Pain (VAS Scale) | Analgesic Intake and Unscheduled Appointments | **1. Measurement of Primary Outcome (Postoperative Pain Reduction):** Pain Assessment Method: Visual Analog Scale (VAS). **2. Measurement of Secondary Outcomes** Analgesic Intake: Patients recorded the number of analgesic tablets taken daily.  **Unscheduled Emergency Visits** Records of patients who required emergency intervention due to severe pain were reviewed. | **Postoperative Pain** (VAS Scale 0–100) Patient self-reported pain scores Pre-op, 1st, 2nd, 3rd, 4th, 5th, **6th, 7th day An**algesic Intake Patient records (Ibuprofen 400 **mg) Daily for 7** days Emergency Appointments Clinical records Up to 7 days post-RCR |

**Table 3. Characteristics of other outcomes**

| First author | Year of publication | Periapical lesion size | Healing rate based on radiographs | Reduction in post-treatment pain | Postoperative pain (VAS score) | Complications |
| --- | --- | --- | --- | --- | --- | --- |
| Zakaria S. et al. | 2024 | Not evaluated | Not evaluated | Not evaluated | Not evaluated | None significant |
| Tamer M. et al. | 2023 | Not evaluated | Not evaluated | Lower reduction in diode laser group | Recorded at each interval | None significant |
| Santos M. et al. | 2019 | Not evaluated | Not evaluated | More significant reduction in PDT group | Reported at all intervals | None significant |
| Maheshwari P. et al. | 2024 | Not evaluated | Not evaluated | Significant reduction in LAI group after 48 and 72 hours | VAS reported at time intervals | None reported |
| Kaplan T. et al. | 2022 | Not evaluated | Not evaluated | Similar between groups, no significant difference | Evaluated on NRS, no significant difference | None reported |
| Ismail H. et al. | 2023 | Not evaluated | Not evaluated | More reduction in the LLLT group | Evaluated at all points | None reported |
| Shah D. et al. | 2021 | Significant reduction in the LLLT group | Better in LLLT group | Less pain in the LLLT group, not significant | Evaluated at all time intervals | None reported |
| Abielhassan M. et al | 2021 | Not specified | Not evaluated | Not evaluated | Not evaluated | None reported |
| Tamer M. et al. | 2024 | Not evaluated | Not evaluated | Yes, especially in groups with combined IAS and LLLT lasers | Significant reduction with laser | None reported |
| Shivangi S. et al. | 2023 | Not measured | Not measured | Significant reduction in Groups 2 and 3 compared to Group 1 | Group 2 and Group 3 had better pain reduction than Group 1 | None reported |
| Morsy D.A. et al. | 2018 | Not evaluated | Not evaluated | Significant reduction in postoperative pain in diode laser group (p < 0.001) | Recorded at multiple intervals | None significant​ |
| Noferesti M. et al. | 2024 | Evaluated (PAI score) | No significant difference between Ca(OH)₂ and Ca(OH)₂ + laser groups | No significant difference at 3 and 6 months | Not evaluated | None significant​ |
| Arslan H. et al. | 2017 | Evaluated (PAI score 2-3) | Not evaluated | Significant reduction in LLLT group for the first 4 days, no difference after day 5 | Recorded at multiple intervals | None significant​ |

**Table 4. Characteristics of intervention and comparator**

| **Study** | **Population** | **Intervention** | **Conventional Therapy (Control)** | **Active control 1** | **Active control 2** | **Outcome** | **Time** | **Instrument** |
| --- | --- | --- | --- | --- | --- | --- | --- | --- |
| Abbara et al. 2023 | Large asymptomatic apical lesions (>5 mm according to the Venskutonis classification ) in maxillary incisors | Diode laser (810 nm) + final irrigants : ( NaOCl 5.25%, EDTA 17%, and CHX 2%) | Lima XP-Endo Finisher + conventional irrigant | Passive ultrasonic irrigation + final irrigants ( NaOCl 5.25%, EDTA 17%, and CHX 2%) |  | Pain | 1, 3, 7 and 14 day weeks | Visual analog scale 10 cm |
| Abbara et al. 2024 | Large asymptomatic apical lesions (>5 mm according to the Venskutonis classification ) in maxillary incisors | irrigant activation system (IAS) within the root canal | Activation of the final irrigants was performed by passive ultrasonic irrigation (PUI) | Irrigant activation was performed with PUI. | IAS Laser + LLLT | Pain | 1, 3, 7 and 14 day weeks | Visual analog scale 10 cm |
| Abielhassan et al. 2021 | Necrotic periapical lesion | Low power diode laser (940 nm) | Conventional irrigation solution of sodium hypochlorite ( NaOCl ) at 1.25% and EDTA at 17% | 0.2% Nanocytosan Irrigation Solution |  | Survival | 6, 12 months | Clinical examination, radiographic evaluation and preoperative CBCT |
|  |  |  |  |  |  | Sensitivity | 6, 12 months | electric pulp tester |
| Arslan et al. | Root canal retreatment. Periapical lesions (mandibular molar teeth) | Low Level Laser Therapy (LLLT) (l=970 ± 15 nm, 14-W) | LLLT (placebo) |  |  | Pain | 1, 2, 3, 4, 5, 6 and 7 days | Visual analog scale 10 cm |
|  |  |  |  |  |  | Number of patients who required analgesics |  |  |
|  |  |  |  |  |  | Consumption of painkillers | Daily tablet log |  |
| Fahim et al. 2024 | Periapical lesion (tooth with single-rooted anterior maxillary pulp necrosis) | Diode + Conventional Irrigation  (940 nm) | Conventional irrigation + Calcium hydroxide ( Ca( OH)2) | Double Laser ( Er,Cr :YSGG /Diode)  Er,Cr :YSGG laser (2780 nm)  Diode laser (940 nm) |  | Bacterial count |  | Colony forming units per milliliter (CFU/ml) |
| Ismail et al. 2023 | Acute or chronic infection with symptomatic apical periodontitis | Laser Activated Irrigation ( LAI) (980 nm wavelength, 2.5 W power, 100 Hz frequency) | Conventional irrigation + Mock Laser simulated laser (no activation) | Low Level Laser Therapy (LLLT) (980 nm, 10 Hz frequency) |  | Pain | 24, 48 and 72 hours | Visual Analog Scale (VAS) (0–10 cm) |
| Kaplan et al. 2022 | Symptomatic apical periodontitis (mandibular molars) | Conventional irrigation followed by 980 nm diode laser irradiation | Conventional irrigation | EDDY1 Sonic Irrigation Activation System... | EDDY Sonic Irrigation Activation System followed by 980 nm Diode Laser Irradiation | Pain | 8, 24, 48 hours and 7 days | Numerical Rating Scale - NRS |
|  |  |  |  |  |  | consumption of painkillers | 8, 24, 48 hours and 7 days |  |
| Maheshwari et al. 2024 | Teeth: Molars with symptomatic apical periodontitis | LAI - Laser-activated irrigation / Canal Disinfection activated using laser therapy) | Conventional irrigation + ML - Mock Laser / Placebo | PBS - Photobiostimulation (buccal and lingual/palatal mucosa) |  | Pain | 24, 48 and 72 hours | Visual Analog Scale (VAS) (0–10 cm) |
| Morsy et al. 2018 | Chronic apical periodontitis | Diode laser (980 nm) | Conventional endodontic treatment | - |  | Antibacterial effect  Pain |  | Numeric Rating Scale (NRS) by categories |
| Shah et al. 2022 | Apical periodontitis (single-rooted permanent teeth) | Conventional root canal therapy + Low Level Laser Therapy (LLLT). Duolase 660 nm, at a power of 100 mW | Conventional root canal therapy + Placebo | - |  | Pain | 0, 7 and 14 days | Modified Visual Analog Scale (VAS) |
|  |  |  |  |  |  | Periapical Healing / Lesion Size Reduction | 3, 6 and 9 months |  |
| Shivangi et al. 2023 | Single-rooted teeth (pulp necrosis and symptomatic apical periodontitis) | Continuous rotary instrumentation + laser irradiation | Continuous rotating instrumentation, without laser irradiation (940 nm diode at 1 W) | Mechanized reciprocating instrumentation + laser irradiation (940 nm diode at 1 W) |  | Pain | 24, 48 and 72 hours | Modified Visual Analog Scale (VAS) |
| Noferesti et al. 2024 | periapical lesions (root canal retreatment) Single-rooted teeth | Laser Irradiation + Ca( OH)2  diode of 980 nm | Conventional irrigation + Calcium hydroxide ( Ca( OH)2) |  |  | Radiographic healing of periapical lesions | 3 and 6 months | Ørstavik Periapical Index (PAI) |
|  |  |  |  |  |  |  |  |  |
